# Vinegar as a Hemostatic

**Published:** 1897-05

**Authors:** C. S. Estep


### VINEGAR AS A HEMOSTATIC.

I wish to confirm the article I saw in your valuable journal a few days ago concerning the use of vinegar as an uterine hemostatic.

My faith is so strong in its favor that I have never used anything else for the past four years.

I used it in a fountain syringe, diluted, equal parts, with hot water. I push the nozzle well up into the womb when the hemorrhage is post-partum.

When there is an abortion on hand and the placenta is loosening and coming away piece at a time, which process, as every physician knows, sometimes requires days to complete, I use intra-uterine injections by means of a fountain syringe and uterine irrigator.

If there is no immediate danger I attempt to clear the uterine cavity by dilating the os and using a curette, but where there is a syncope and a general anemic condition this procedure might prove fatal, and in such cases I use my injection and place a tight tampon.

In all cases (and there are a great many I might relate, but it would require too much space) I have returned inside of twenty-four hours and found my patient quiet, free from pain, with a good pulse, etc., and on removing the tampon found the fragments outside of the cervix and no hemorrhage except a small black clot.

Cessation of the pains is an indication for removing the tampon. Afterwards I use injections of only hot water.

In post-partum hemorrhages I do not use a tampon, but will repeat the injection if necessary.—DR. C. S. ESTEP, in *Med. Council.*

